# Prevalence of short and long sleep duration: Ravansar NonCommunicable Disease (RaNCD) cohort study

**DOI:** 10.1186/s12889-022-14061-4

**Published:** 2022-08-29

**Authors:** Arezu Najafi, Samaneh Akbarpour, Farid Najafi, Roya Safari-Faramani, Khosro Sadeghniiat-Haghighi, Faezeh Aghajani, Samaneh Asgari, Forugh Aleebrahim, Amin Nakhostin-Ansari

**Affiliations:** 1 Occupational Sleep Research Center, Tehran University of Medical Sciences, Tehran, Iran; 2 Sleep Breathing Disorders Research Center, Tehran University of Medical Sciences, Tehran, Iran; 3 Department of Epidemiology, School of Health, Research Center for Environmental Determinants of Health, Research Institute for Health, Kermanshah University of Medical Sciences, Kermanshah, Iran; 4 Research Development Center, Arash Women’s Hospital, Tehran University of Medical Sciences, Tehran, Iran; 5 Prevention of Metabolic Disorders Research Center, Research Institute for Endocrine Sciences, Shahid Beheshti University of Medical Sciences, Tehran, Iran; 6 School of Health, Isfahan University of Medical Sciences, Isfahan, Iran; 7 Sports Medicine Research Center, Neuroscience Institute, Tehran University of Medical Sciences, Tehran, Iran; 8 Neuromusculoskeletal Research Center, Iran University of Medical Sciences, Tehran, Iran

**Keywords:** Epidemiology, Iran, Sleep hygiene

## Abstract

**Background:**

Prevalence of short and long sleep duration varies in different countries and changes over time. There are limited studies on Iranians’ sleep duration, and we aimed to evaluate the prevalence of short and long sleep duration and associated factors among people living in Kermanshah, Iran.

**Methods:**

This population-based cross-sectional study was conducted between November 2014 and February 2017. Data was collected from 10,025 adults aged 35 to 65 years using census sampling, and we evaluated the short and long sleep duration (≤ 6 and ≥ 9 h, respectively) and its relation with the socio-demographic factors and health-related status of the participants.

**Results:**

Mean age of participants was 48.1 years (standard deviation = 8.2), and 47.4% of participants were male. Of our participants, 11.6% had short, and 21.9% had long sleep duration. Age ≥ 50 years, female gender, being single, mobile use for longer than 8 h per day, working in night shifts, moderate and good levels of physical activity, BMI ≥ 30, past smoking, and alcohol use were associated with short sleep duration (*P* < 0.05). Female gender and living in rural areas were associated with long sleep duration (*P* < 0.05).

**Conclusion:**

In the Ravansar population, short and long sleep duration are prevalent, with long sleep duration having higher prevalence. People at risk, such as night shift workers, as well as modifiable factors, such as mobile phone use, can be targeted with interventions to improve sleep hygiene.

**Supplementary Information:**

The online version contains supplementary material available at 10.1186/s12889-022-14061-4.

## Introduction

Ample evidence states that sleep duration is the most crucial sleep component which affects an individual’s health [1]. Either inadequate or excessive sleep can have adverse effects on health and are associated with an increased risk of mortality [2–4]. Insufficient sleep is independently associated with obesity [5] and hypertension [6]. On the other hand, long sleep duration is associated with metabolic syndrome [7]. Also, short and long sleep durations are associated with worse cardiovascular health, increased risk of diabetes development, increased risk of stroke, and mortality due to stroke [8–10].

There are controversies regarding the definition of short and long sleep durations in the literature. In epidemiological studies, habitual sleep for six hours or less is the suggested definition for short sleep duration [11]. On the other hand, long sleepers are individuals with a habitual sleep of 9 h or more [12]. The prevalence of long sleep duration is estimated to be between 24% to 38.4% in different countries [13]. Short sleep duration is less prevalent throughout the world, and it affects between 0.8% to 10.1% of people in different studies [13]. Plenty of studies have also been conducted on the factors associated with abnormal sleep duration, and several factors, such as age, employment status, marital status, educational level, and race, have been found to affect sleep duration [14, 15]. Associations of sleep disorders and sleep duration with socio-demographic and clinical factors have been studied in some countries [16–18]. However, their findings may not apply to all other countries, particularly developing countries [19].

Several studies have evaluated the prevalence of abnormal sleep duration and other sleep disorders in university students and Children [20–23] in Iran, but limited studies have been conducted on the prevalence of abnormal sleep duration among the general Iranian population. In a study in Fasa, Yazdanpanah et al. found that 19.5% of participants slept for less than six hours, and short sleep duration was more common among smokers, older adults, and males [20]. In another study by Najafian et al., 30.3% of people in Isfahan slept for less than 6 h, and 8.7% slept for more than 9 h [21]. Even though these two studies were large-scale studies giving valuable preliminary information about abnormal sleep duration status in Iran, it was not the main objective in both studies. They were conducted to evaluate the association between sleep duration and cardiometabolic risk factors.

To summarize, short and long sleep durations are risk factors for several health conditions. Identifying factors associated with abnormal sleep duration is essential to inform early identification, intervention, and even prevention in the high-risk population. Studies evaluating prevalence and factors associated with short and long sleep durations are limited in Iran. Therefore, we aimed to evaluate the prevalence of short and long sleep duration and associated factors in a large-scale study in Revansar, Iran.

## Methods

### Study participants

The present study is based on the Ravansar Non-Communicable Disease (RaNCD) Cohort Study data, a part of the national cohort study of the Prospective Epidemiologic Research Studies of Iranian Adult (PERSIAN). The details of this cohort study were reported in another publication [22]. This study was conducted on rural and urban people living in the Ravansar region in Kermanshah province, Iran’s western part. Altogether, 15,000 people between 35–65 years old live in the Ravansar region, and most of them are of Kurdish ethnicity and speak Kurdish. The sampling method of this study was census sampling, and in total, 10,065 adults between 35–65 years old agreed to participate in this study.

A trained research assistant performed a door-to-door survey in the urban area and explained the study goals and objectives to households. Those who consented to participate in the study were registered and given a code. A date was scheduled for them to attend the cohort center for evaluations. Health centers performed the same procedure in rural areas to enroll people. Data collection was performed between November 2014 and February 2017 by trained interviewers, with a response rate of 93.3% [22]. In the present study, 40 participants were excluded due to lack of sleep data, and finally, 10,025 participants entered the study. We took informed consent from all participants. All study procedures were performed according to the declaration of Helsinki.The study protocol was approved by the Ethics Committee of the Kermanshah University of Medical Sciences.

### Measurements

Educated local staff collected data from people’s homes. All participants received a reminder on the day before the sampling to be present at home on the census day. They were also told that they needed to be fasting at the sampling time.

The collected data consisted of socio-demographic characteristics (i.e.age, gender, and educational level), physical activity status, alcohol and tobacco use, and past medical history. Each family’s assets, including homeownership, room per capita, having dishwasher, freezer, vacuum cleaner, laundry machine, personal computer, internet access, motorcycle, TV, cellphone, and car, were measured to assess their economic status. There was a score defined for each subject, and the Principal Component Analysis (PCA) method was used to estimate the Wealth Index, which is a reliable method for this evaluation [23]. The wealth index was changed to a quintile variable by PCA, and all participants were divided into five groups. This variable was used as a surrogate of individuals’ economic status.

We asked participants how many hours they spent a day on their cellphones calling, answering calls, texting, gaming, and surfing on the internet to evaluate their cellphone use. They were classified into three groups of low (less than 3 h), moderate (between 3 to 7.9 h), and high (8 h and longer) users based on their cellphone use time.

Based on the United States National Health Interview Survey (NHIS), the participants’ smoking status was categorized as smoking at present, had smoked before, and never smoked [24]. As the prevalence of alcohol consumption in Iran is low, we decided to evaluate lifetime alcohol consumption [25]. So, the participants were asked if they had had a drink during their life to evaluate alcohol consumption. Recalled data on smoking and alcohol consumption used in the current study are both reliable and valid measurement methods [26–28].

Questions on physical activity contained three parts of physical activity during work time, leisure time, and sports. The time spent on physical activity during the week was asked, and metabolic equivalents (METs) were measured according to that. Based on METs, participants were divided into three groups of low physical activity (24–36.5), moderate physical activity (36.6–44.9), and good physical activity (equal and more than 45).

The Body Mass Index (BMI) was calculated using each participant’s measured weight and height and categorized into three groups of less than 25, 25 to 29, and 30 or more. Participants’ waist circumference (WC) was measured at the level of the umbilicus.

To measure participants’ blood pressure, they were asked to sit on a chair and relax for 10 min. Blood pressure was measured using the right arm. Participants’ blood pressure was measured in two consequent times. There was a 10-min interval between the measures, and participants were asked to stay calm between the measures. The mean of two blood pressure readings was recorded. Having systolic blood pressure over 140 mmHg or diastolic blood pressure over 90 mmHg or consumption of antihypertensive medications was considered high blood pressure [29].

Participants with fasting blood sugar of 126 mg/dL and over or those using medications for diabetes were considered diabetic. Those with total cholesterol of more than 240 mg/dL or those consuming medications for hyperlipidemia were considered to have hyperlipidemia.

Expert psychologists used self-administrated questionnaires and clinical examinations to divide participants as depressed and not depressed based on the Diagnostic and Statistical Manual of Mental Disorders, 4th Edition criteria [30] to evaluate current depression status [31].

Participants were asked how many hours they sleep during a normal day, and based on their answers were categorized those who sleep for six hours or less, those who sleep between 6 to 9 h, and those who sleep for 9 h or more, which are considered as short, normal, and long sleep duration, respectively [11, 12, 32].

### Statistical analysis

We used mean and standard deviation (SD) and number and percentage to describe quantitative and categorical variables. A chi-squared test was used to compare qualitative variables. T-test and one-way ANOVA tests were used to compare two and more than two groups in terms of quantitative variables, respectively. In the end, to assess the relationship between variables and the desired outcome, the adjusted odds ratio was calculated in multinomial logistic regression.

About 2.11% of the data on variables was missing. These missing data were replaced using the single imputation method and the regression model of the mice package of the R software [33].

All analyses were done with the STATA software, and P less than 0.05 was considered statistically significant.

## Results

**Fig. 1:**
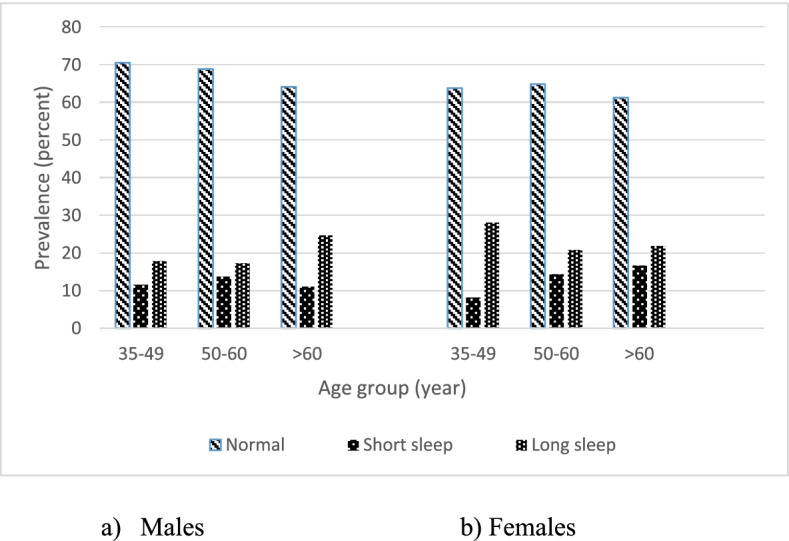
Prevalence of short, long, and normal sleep duration based on age and gender. **a** Males. **b** Females

In total, 10,025 people participated in the study with a mean age of 48.1 years (SD = 8.24, range: 35–65), of which 4753 (47.41%) were male. Table 1 shows the socio-demographic and health-related characteristics of the participants. As it is shown in Table 1, 1164 participants (11.61%) had short, and 2198 (21.92%) had long sleep duration. Gender distribution in those with normal and short sleep duration was not significantly different (*P* = 0.943); however, most individuals with long sleep duration were females, and gender distribution was significantly different in those with long sleep duration compared to those with normal (*P* = 0.003) and short sleep duration (*P* < 0.001). The prevalence of short and long sleep durations based on age and gender is shown in Fig. 1.

**Table 1:** Socio-demographic and health-related characteristics of participants based on self-reported sleep duration- in Iranian adults—Ravansar Non-Communicable Disease (RaNCD) cohort study

	Self-reported total sleep time (hr)				
Variables	Total (*n* = 10,025)	≤ 6 h (*n* = 1164)	6–9 h (*n* = 6663)	≥ 9 h (*n* = 2198)	*P*-value
**Age (years)**	48.10 (8.24)	49.57 (8.07)	48.03 (8.12)	47.55 (8.61)	** < 0.0001**
**Gender**					
Male	4753 (47.4%)	578 (49.7%)	3301 (49.5%)	874 (39.8%)	** < 0.0001**
Female	5272 (52.6%)	586 (50.3%)	3362 (50.5%)	1324 (60.2%)	
**Education**					
Lower than diploma	8295 (82.7%)	980 (84.2%)	5394 (80.9%)	1921 (87.4%)	** < 0.0001**
Diploma	967 (9.6%)	110 (9.4%)	670 (10.1%)	187 (8.5%)	
Higher than diploma	763 (7.6%)	74 (6.4%)	599 (9%)	90 (4.1%)	
**Marital status**					
Married	9044 (90.2%)	1043 (89.6%)	6088 (91.4%)	1913 (87%)	** < 0.0001**
Single	981 (9.8%)	121 (10.4%)	575 (8.6%)	285 (13%)	
**Residence**					
Urban	5942 (59.3%)	704 (60.5%)	4063 (61%)	1175 (53.5%)	** < 0.0001**
Rural	4083 (40.7%)	460 (39.5%)	2600 (39%)	1023 (46.5%)	
**Wealth index status**					
The poorest	1999 (19.9%)	231 (19.8%)	1240 (18.6%)	528 (24%)	** < 0.0001**
Second poor	2005 (20%)	258 (22.2%)	1252 (18.8%)	495 (22.5%)	
Middle	2009 (20%)	238 (20.5%)	1325 (19.9%)	446 (20.3%)	
Second rich	2010 (20%)	231 (19.8%)	1400 (21%)	379 (17.3%)	
The richest	2002 (20%)	206 (17.7%)	1446 (21.7%)	350 (15.9%)	
**Mobile use time (hours/day)**	5.8 (4.57)	6.02 (4.64)	5.99 (4.56)	5.24 (4.52)	** < 0.0001**
**Night shift worker**					
Yes	1179 (11.8%)	184 (15.8%)	808 (12.1%)	187 (8.5%)	** < 0.0001**
No	8846 (88.2%)	980 (84.2%)	5855 (87.9%)	2011 (91.5%)	
**BMI (kg/m2)**	27.59 (5.88)	27.61 (4.75)	27.640 (6.41)	27.45 (4.63)	0.135
**WC (cm)**	97.27 (10.80)	97.62 (11.24)	97.39 (10.67)	96.73 (10.93)	**0.024**
**Physical activity**					
Low	2759 (27.5%)	206 (17.7%)	1685 (25.3%)	868 (39.5%)	** < 0.0001**
Moderate	5153 (51.4%)	601 (51.6%)	3507 (52.6%)	1045 (47.5%)	
Good	2113 (21.1%)	357 (30.7%)	1471 (22.1%)	285 (13%)	
**Smoking status**					
Nonsmokers	8021 (80%)	873 (75%)	5341 (80.1%)	1807 (82.2%)	** < 0.0001**
Current smokers	1175 (11.7%)	159 (13.7%)	783 (11.8%)	233 (10.6%)	
Past smokers	829 (8.3%)	132 (11.3%)	539 (8.1%)	158 (7.2%)	
**Alcohol use**					
Yes	629 (6.4%)	103 (8.8%)	421 (6.3%)	105 (4.8%)	** < 0.0001**
No	9396 (93.6%)	1061 (91.2%)	6242 (93.7%)	2093 (95.2%)	
**Depression**	324 (3.2%)	45 (3.9%)	196 (2.9%)	83 (3.8%)	0.068
**Cardiovascular diseases**	1366 (13.6%)	192 (16.5%)	886 (13.3%)	288 (13.1%)	**0.01**
**Hypertension**	1576 (15.7%)	206 (17.7%)	1049 (15.7%)	321 (14.6%)	0.064
**Hyperlipidemia**	4450 (44.4%)	510 (43.8%)	2990 (44.9%)	950 (43.2%)	0.367
**Diabetes**	822 (8.2%)	113 (9.7%)	513 (7.7%)	196 (8.9%)	**0.027**

*BMI* Body mass index, *WC* Waist circumferences

Values are expressed as frequency (percent) except for age, mobile use time, BMI, and WC, which are expressed as mean (SD)

People with healthy sleep duration had higher educational levels and wealth index scores than those with abnormal sleep duration (*P* < 0.0001). Also, the percentage of married individuals and individuals living in urban areas was significantly higher in this group than those with abnormal sleep duration (*P* < 0.0001). Although BMI was not significantly different between people with different sleep durations (*P* = 0.135), WC was larger in those with shorter sleep duration (*P* = 0.024). Good physical activity was more common among people with short sleep duration, and low physical activity was more prevalent in those with long sleep duration (*P* < 0.0001). Prevalence of current or past smoking was more in people with short sleep duration compared to other groups (*P* < 0.0001). Also, the percentage of nonsmokers and nonalcohol users were more in people with long sleep duration (*P* < 0.0001). Compared to those with healthy sleep durations, diabetes was more prevalent in short and long sleepers (*P* = 0.027). Cardiovascular diseases were more common among those with shorter sleep duration than others (*P* = 0.01). Gender-specefic analyses are also presented in Supplementary Tables 1 and 2.

The results of multinomial regression are shown in Table 2. Older ages (Age:51–60 and ≥ 60, OR: 1.46 and 1.71, respectively), female gender (OR 1.33), being unmarried (OR: 1.31), mobile use time ≥ 8 h (OR:1.31), working in night shifts (OR:1.34), obesity (OR:1.21), moderate physical activity (OR:1.48), good physical activity (OR:2.18), past smoking (OR:1.31), and drinking alcohol (OR:1.46) were significant for short sleep duration (all *P* < 0.05). Likewise, there was a marginally significant relationship between rural area residency (OR: 0.88; *P* = 0.076) and depression (OR: 0.74; *P* = 0.087) with short sleep duration.

**Table 2:** Adjusted Odds ratios (OR) and 95% confidence intervals (CI) from full multinomial logistic regression for the relation between the covariates and self-reported sleep hours in Iranian adults—Ravansar Non-Communicable Disease (RaNCD) cohort study

	Short sleep duration (≤ 6 h)versus normal sleep duration (6–9 h)			Long sleep duration (≥ 9 h)versus normal sleep duration (6–9 h)		
Variables	Odds ratio	95% CI	*p*-value	Odds ratio	95% CI	*p*-value
**Age (years)**						
≤ 50	1			1		
51–60	1.46	1.26–1.70	** < 0.0001**	0.75	0.66–0.85	** < 0.0001**
≥ 60	1.71	1.38–2.11	** < 0.0001**	0.78	0.66–0.93	**0.006**
**Gender (male gender is reference)**	1.33	1.09–1.62	**0.005**	1.16	1.00–1.36	**0.049**
**Education**						
Lower than diploma	1			1		
diploma	1.12	0.88–1.41	0.334	0.82	0.67–0.99	**0.041**
Higher than diploma	1.04	0.77–1.40	0.784	0.40	0.30–0.52	** < 0.0001**
**Marital status (married is ref)**	1.31	1.05–1.63	**0.015**	1.13	0.96–0.133	0.122
**Residence (urban is ref)**	0.88	0.76–1.01	0.076	1.31	1.18–1.45	** < 0.0001**
**Wealth index status**						
The poorest	1			1		
Second poor	1.12	0.92–1.36	0.247	0.93	0.80–1.08	0.393
Middle	0.97	0.79–1.19	0.795	0.82	0.70–0.95	**0.013**
Second rich	0.92	0.74–1.13	0.459	0.65	0.55–0.76	** < 0.0001**
The richest	0.85	0.66–1.08	0.184	0.68	0.57–0.83	** < 0.0001**
**Mobile use time (hours)**						
< 3^a^	1			1		
3–7.9	1.13	0.95–1.35	0.146	0.96	0.84–1.09	0.555
≥ 8	1.31	1.05–1.64	**0.013**	0.94	0.79–1.12	0.503
**Night shift workers**	1.34	1.10–1.64	**0.003**	0.79	0.65–0.94	**0.012**
**BMI (kg/m2)**						
< 25^a^	1			1		
25–29.9	1.01	0.85–1.16	0.958	0.97	0.86–1.09	0.682
≥ 30	1.21	1.02–1.45	**0.029**	0.87	0.76–1.01	0.066
**Physical activity**						
Low^a^	1			1		
Moderate	1.48	1.24–1.76	** < 0.0001**	0.45	0.40–0.51	** < 0.0001**
Good	2.18	1.78–2.66	** < 0.0001**	0.29	0.25–0.35	** < 0.0001**
**Smoking status**						
Nonsmokers ^a^	1			1		
Current smokers	1.14	0.93–1.41	0.191	1.00	0.83–1.19	0.997
Past smokers	1.31	1.05–1.63	**0.015**	1.01	0.82–1.22	0.957
**Alcohol use**	1.46	1.14–1.87	**0.003**	0.86	0.67–1.09	0.232
**Depression**	0.74	0.53–1.04	0.087	0.87	0.65–1.13	0.295
**Cardiovascular diseases**	1.17	0.96–1.44	0.115	0.94	0.78–1.11	0.453
**Hypertension**	0.91	0.74–1.11	0.350	0.86	0.72–1.01	0.082
**Diabetes**	1.17	0.93–1.46	0.163	1.23	1.02–1.47	**0.024**

^a^ Reference group

Regarding long sleep duration, we found a significant odds ratio for older ages (51–60 years and ≥ 60 years, OR: 0.75 and 0.78, respectively), female gender (OR: 1.16), education level of diploma (graduation from high school) and higher than diploma (OR: 0.82, 0.40, respectively), rural area residency (OR:1.31), higher economic levels (middle, second rich, richest, OR: 0.82, 0.65,0.68, respectively), working in night shifts (OR:0.79), moderate physical activity (OR:0.45), higher physical activity (OR:0.29) and diabetes (OR: 1.23) in multivariate analysis (all *P* < 0.05). Likewise, there were marginally significant relationships between obesity (OR: 0.87; *P* = 0.066) and hypertension (OR:0.86; *P* = 0.082) with long sleep duration.

## Discussion

This study was the first population-based study in Iran evaluating the prevalence of short and long sleep duration and related risk factors. We found that 11.6% of our participants had short and 21.9% had long sleep duration. Age ≥ 50 years, female gender, single marital status, mobile use for longer than 8 h, working night shifts, moderate and good levels of physical activity, BMI ≥ 3, past smoking, and alcohol use were associated with short sleep duration. Female gender and living in rural areas were associated with long sleep duration.

In another study, Najafian et al. (2019) evaluated the relation between sleep duration and hypertension in Isfahan, Iran [34]. In their study, 8.7% of participants had long sleep duration (≥ 9 h), which is approximately half of the prevalence of long sleep duration in our study, and 30.3% had short sleep duration (≤ 6 h), which is three folds compared to our research. Sleep patterns seem entirely different in the two studies considering these differences. Differences in these populations’ socio-demographic characteristics and health-related conditions may be other reasons for the difference in sleep duration of these two populations.

The prevalence of short sleep duration was 11.6% in our study, which is much higher compared to other countries such as the United Kingdom (10.1%), Sweden (4.7%), Norway (5.6%), and Netherland (0.8%) [13]. In contrast, long sleep duration is more prevalent in developed countries such as the United Kingdom (25.5%), Sweden (30.1%), Norway (26.3%), and Netherland (25.7%) compared to our study (21.9%). The prevalence of short sleep duration was reported to be 33.8% in Saudi Arabia by Ahmed et al. (2017). Mean sleep time was 6.4 h in their study [35]. It seems that sleep duration is shorter in developing countries compared to developed countries. There are differences between socioeconomic status and peoples’ lifestyles in these countries. We found that worse economic status and lower educational level are related to longer sleep duration in our study. Vicky et al. (2018) found that an educational level of less than secondary school is related to long sleep duration among adults (OR = 3.25, 95% CI = 9.04). Low household income was also related to long sleep duration among pre-school kids (OR = 2.59, CI = 1.12–5.65) [36]. In contrast, Felden et al. (2015) reported that poor socioeconomic status is related to shorter sleep durations and worse sleep quality [37]. Considering these findings, different lifestyles in developed and developing countries may contribute more to people’s longer sleep duration as lower socioeconomic status is not necessarily related to short sleep duration.

The prevalence of long sleep duration is relatively high in Ravansar, indicating a need for interventions for high-risk populations. Living in rural areas, lower educational level, lower wealth score index were related to long sleep duration in our study. This finding is supported by previous studies in other populations, as poor socioeconomic status and lower educational levels were associated with long sleep duration [17, 38, 39]. There is a reverse association between sleep duration and waking activities, and more activities by those with better socioeconomic status and more educated people. Their possibly more efforts for educational and career developments and achievements may explain the lower prevalence of long sleep duration in these groups [38, 40]. Lifestyle and factors associated with occupational and economic activities may be reasons explaining the longer sleep duration in people living in rural areas [39]. In total, people with lower socioeconomic status and those living in rural areas can be targeted for interventions designed to reduce the prevalence of long sleep duration.

Female gender was associated with both short or long sleep durations in our study. Various studies indicate different results regarding the propensity of the female gender to short and long sleep duration [14, 41]. However, in this cohort, the studied population share cultural and behavioral issues that may lead to the same phenotype for a specific risk factor; association of female gender with both long and short sleep duration may be due to heterogeneity in several risk factors that we have not evaluated yet in this population. Certain behaviors or cultural beliefs among women that lead to different sleep durations might be the cause. There is a need for further studies on this issue; however, whatever the cause is, females may benefit from interventions to improve sleep duration.

We found that different sedentary, such as mobile phone use, and non-sedentary behaviors, such as shift working and higher physical activity levels, are associated with shorter sleep durations. It is suggested in the previous studies that both sedentary and non-sedentary activities may affect sleep opportunities, leading to short sleep duration and its physical and mental consequences [42]. Using a mobile phone for eight hours/day or longer was related to short sleep duration in our study (OR = 1.31, 95% CI = 1.05–1.64). This is in line with Tamura et al.’s study, as using mobile phones for 5 h/day or longer was related to insomnia in their study (OR = 4.27, 95% CI = 1.5–12.16) [43]. Using excessive and bedtime use of the mobile phone is not only related to short sleep duration, but also it can reduce sleep quality and increase alertness near the sleep episode [44]. These findings suggest that increasing usage of mobile phones in communities can seriously affect sleep quality and sleep duration, and there is a need for interventions to change this trend. Mobile phone use is a modifiable risk factor to be a feasible intervention target.

Heavier physical activity among our participants was associated with shorter sleep duration (*P* < 0.0001). In previous studies, regular and acute exercise was related to longer sleep duration [45], contrary to our findings. Our study’s total physical activity included physical activity during work time, leisure time, and doing sports. Higher physical activity levels in our study may be due to various activities and not only exercise and doing sports. People who have heavier jobs will have higher METs, and they may have less time to sleep and relax. Putting all together, doing sports and exercise can improve sleep duration and quality. However, working for long hours and other types of activity may relate to short sleep duration. Future studies are needed to determine the relationship between different activities and sleep duration.

Long sleep duration was associated with diabetes in our study, which is in line with previous studies [45]. However, there was no other association between abnormal sleep duration and cardiovascular disease, hypertension, and hyperlipidemia. In a study with a sample size of 700,000 individuals, Grandner et al. in 2016 found that hypertension is more prevalent among those with short or long time duration than those with normal sleep duration [46]. Grandner et al. also found that the relation between short sleep duration and hypertension is stronger. Short sleep duration was related to a higher risk of metabolic syndrome [47], hyperlipidemia [48], and diabetes [49] in various studies. Previous studies have demonstrated lower HDL cholesterol levels and higher Triglycerides levels in those with short sleep duration [50]. There may be other confounding factors that have concealed the effects of sleep duration on the cardiometabolic conditions of our participants. Detailed evaluation of cardiometabolic risk factors and their relations with sleep duration in the Iranian population can better understand this phenomenon among Iranians.

### Limitations

There are several limitations to our study. First, the Ravansar population may be different from other cities in Iran in terms of both culture and socioeconomic status. The lifestyle in industrial cities is different from a Ravansar, a mountainous area with Kurdish inhabitants. As a result, our study may not be applicable to other regions in Iran. Future studies are needed to evaluate the sleep duration and its relation with socio-demographic characteristics in other cities in Iran. Although we conducted this study as part of the PERSIAN cohort, we did not have a chance to follow the participants. Accordingly, all health-related exposures are prevalent conditions (long-term conditions with or without treatment) that are different from incident events. Following participants through years can guide us to understand the exact effects of socio-demographic characteristics on sleep duration better. Another limitation of our study was that we relied on participants’ self-report of sleep duration, socio-demographic status, and health-related variables, which may lead to reporting bias as people may report shorter or longer times. We also did not evaluate the frequency of alcohol consumption in our study, which is another limitation of this study. Finally, sleep disorders such as insomnia and restless legs, which may contribute to short sleep duration, were not evaluated in our study.

## Conclusion

Prevalence of short and long sleep duration is relatively high among the Ravansar population, with more prevalence in the group with long sleep duration. Higher levels of physical activity and mobile phone use may be contributing to short sleep as the time taken to these activities is very possibly impacting upon sleep opportunity. Shift workers are also at the risk of short sleep duration, and may benefit from interventions in this regard. Considering the adverse effects of both short and long sleep duration and the high prevalence of both conditions in our population, we recommend further observational studies on abnormal sleep duration in this population.

## Supplementary Information

**Additional file 1: Supplementary Table 1.** Socio-demographic and health-related characteristics of participants based on self-reported sleep duration in male participants. **Supplementary Table 2.** Socio-demographic and health-related characteristics of participants based on self-reported sleep duration in female participants.

## Data Availability

The data that support the findings of this study are available from the corresponding author, ANA, upon reasonable request.
