# Supplementary material for: Prevalence of short and long sleep duration: Ravansar NonCommunicable Disease (RaNCD) cohort study
Source: BMC Public Health. 2022 Aug 29;22:1631. doi: 10.1186/s12889-022-14061-4 (PMC9422113; doi:10.1186/s12889-022-14061-4)
Supplement: Supplementary file 1 — Additional file 1: Supplementary Table 1. Socio-demographic and health-related characteristics of participants based on self-reported sleep duration in male participants. Supplementary Table 2. Socio-demographic and health-related characteristics of participants based on self-reported sleep duration in female participants. [file 12889_2022_14061_MOESM1_ESM.docx]

| **Supplementary table 1:** Socio-demographic and health-related characteristics of participants based on self-reported sleep duration in male participants | | | | | |
| --- | --- | --- | --- | --- | --- |
|  | Self-reported total sleep time (hr) | | | |  |
| Variables | Total  (n=4753) | ≤6 hr.  (n=578) | 6-9 hr  (n=3301) | ≥9 hr  (n=874) | P-value |
| **Age (years)** | 47.81 (8.04) | 47.98 (7.78) | 47.62 (7.92) | 48.42 (8.66) | **0.028** |
| **Education** |  |  |  |  |  |
| Lower than diploma | 3383 (71.2%) | 424 (73.4%) | 2266 (68.6%) | 693 (79.3%) | **<0.001** |
| Diploma | 751 (15.8%) | 90 (15.6%) | 540 (16.4%) | 121 (13.8%) |  |
| Higher than diploma | 619 (13%) | 64 (11.1%) | 495 (15%) | 60 (6.9%) |  |
| **Marital status** |  |  |  |  |  |
| Married | 4691 (97.2%) | 553 (95.7%) | 3219 (97.5%) | 847 (96.9%) | 0.127 |
| Single | 134 (2.8%) | 25 (4.3%) | 82 (2.5%) | 27 (3.1%) |  |
| **Residence** |  |  |  |  |  |
| Urban | 2921 (61.5%) | 361 (62.5%) | 2084 (63.1%) | 476 (54.4%) | **<0.001** |
| Rural | 1832 (38.5%) | 217 (37.5%) | 1217 (36.9%) | 398 (45.6%) |  |
| **Wealth index status** |  |  |  |  |  |
| The poorest | 624 (13.1%) | 86 (14.9%) | 395 (12%) | 143 (16.4%) | **<0.001** |
| Second poor | 832 (17.5%) | 115 (19.9%) | 530 (16.1%) | 187 (21.4%) |  |
| Middle | 967 (20.3%) | 113 (19.6%) | 679 (20.6%) | 175 (20%) |  |
| Second rich | 1069 (22.5%) | 131 (22.7%) | 756 (22.9%) | 182 (20.8%) |  |
| The richest | 1261 (26.5%) | 133 (23%) | 941 (28.5%) | 187 (21.4%) |  |
| **Mobile use time (hours/day)** | 8.98 (3.78) | 9.09 (3.63) | 9.04 (3.7) | 8.71 (4.11) | 0.06 |
| **Night shift worker** |  |  |  |  |  |
| Yes | 1141 (24%) | 177 (30.6%) | 783 (23.7%) | 181 (20.7%) | **<0.001** |
| No | 3612 (76%) | 401 (69.4%) | 2518 (76.3%) | 693 (79.3%) |  |
| **BMI (kg/m2)** | 26.38 (4.53) | 26.4 (4.16) | 26.43 (4.73) | 26.18 (3.98) | 0.34 |
| **WC (cm)** |  |  |  |  |  |
| <102 | 3465 (72.9%) | 421 (72.8%) | 2406 (72.9%) | 638 (73%) | 0.997 |
| ≥102 | 1288 (27.1%) | 157 (27.2%) | 895 (27.1%) | 236 (27%) |  |
| **Physical activity** |  |  |  |  |  |
| Low | 1579 (33.2%) | 131 (22.7%) | 1065 (32.3%) | 383 (43.8%) | **<0.001** |
| Moderate | 1588 (33.4%) | 202 (34.9%) | 1114 (33.7%) | 272 (31.1%) |  |
| Good | 1586 (33.4%) | 245 (42.4%) | 1122 (34%) | 219 (25.1%) |  |
| **Smoking status** |  |  |  |  |  |
| Nonsmokers | 3026 (63.7%) | 326 (56.4%) | 2149 (65.1%) | 551 (63%) | **0.001** |
| Current smokers | 1068 (22.5%) | 150 (26%) | 711 (21.5%) | 207 (23.7%) |  |
| Past smokers | 659 (13.9%) | 102 (17.6%) | 441 (13.4%) | 116 (13.3%) |  |
| **Alcohol use** |  |  |  |  |  |
| Yes | 625 (13.1%) | 103 (17.8%) | 418 (12.7%) | 104 (11.9%) | **0.002** |
| No | 4128 (86.9%) | 475 (82.2%) | 2883 (87.3%) | 770 (88.1%) |  |
| **Depression** | 74 (1.6%) | 12 (2.1%) | 47 (1.4%) | 15 (1.7%) | 0.462 |
| **Cardiovascular diseases** | 477 (10%) | 73 (12.6%) | 309 (9.4%) | 95 (10.9%) | **0.036** |
| **Hypertension** | 611 (12.9%) | 77 (13.3%) | 414 (12.5%) | 120 (13.7%) | 0.607 |
| **Hyperlipidemia** | 2530 (53.2%) | 301 (52.1%) | 1745 (52.9%) | 484 (55.4%) | 0.349 |
| **Diabetes** |  |  |  |  |  |
| Values are expressed as frequency (percent) except for age, mobile use time, and BMI, which are expressed as mean (SD)  BMI= body mass index; WC= waist circumferences | | | | | |

| **Supplementary table 2:** Socio-demographic and health-related characteristics of participants based on self-reported sleep duration in female participants | | | | | |
| --- | --- | --- | --- | --- | --- |
|  | Self-reported total sleep time (hr) | | | |  |
| Variables | Total  (n=5272) | ≤6 hr  (n=586) | 6-9 hr  (n=3362) | ≥9 hr  (n=1324) | P-value |
| **Age (years)** | 48.37 (8.42) | 51.14 (8.06) | 48.44 (8.31) | 46.97 (8.53) | **<0.001** |
| **Education** |  |  |  |  |  |
| Lower than diploma | 4912 (93.2%) | 556 (94.9%) | 3128 (93%) | 1228 (92.7%) | 0.067 |
| Diploma | 216 (4.1%) | 20 (3.4%) | 130 (3.9%) | 66 (5%) |  |
| Higher than diploma | 144 (2.7%) | 10 (1.7%) | 104 (3.1%) | 30 (2.3%) |  |
| **Marital status** |  |  |  |  |  |
| Married | 4425 (84%) | 490 (83.6%) | 2869 (85.4%) | 1066 (80.5%) | **<0.001** |
| Single | 847 (16%) | 96 (16.4%) | 493 (14.6%) | 258 (19.5%) |  |
| **Residence** |  |  |  |  |  |
| Urban | 3021 (57.4%) | 343 (58.5%) | 1979 (58.9%) | 699 (52.8%) | **0.001** |
| Rural | 2251 (42.7%) | 243 (41.5%) | 1383 (41.1%) | 625 (47.2%) |  |
| **Wealth index status** |  |  |  |  |  |
| The poorest | 1375 (26.1%) | 145 (24.7%) | 845 (25.1%) | 385 (29.1%) | **0.001** |
| Second poor | 1173 (22.2%) | 143 (24.4%) | 722 (21.5%) | 308 (23.3%) |  |
| Middle | 1042 (19.8%) | 125 (21.3%) | 646 (19.2%) | 271 (20.5%) |  |
| Second rich | 941 (17.8%) | 100 (17.1%) | 644 (19.2%) | 644 (19.2%) |  |
| The richest | 741 (14.1%) | 73 (12.5%) | 505 (15%) | 505 (15%) |  |
| **Mobile use time (hours/day)** | 2.98 (3.13) | 2.98 (3.35) | 2.99 (3.1) | 2.95 (3.09) | 0.913 |
| **Night shift worker** |  |  |  |  |  |
| Yes | 38 (0.7%) | 7 (1.2%) | 25 (0.7%) | 6 (0.5%) | 0.203 |
| No | 5234 (99.3%) | 579 (98.8%) | 3337 (99.3%) | 1318 (99.5%) |  |
| **BMI (kg/m2)** | 28.69 (6.69) | 28.8 (5) | 28.82 (7.52) | 28.29 (4.83) | **0.046** |
| **WC (cm)** |  |  |  |  |  |
| <88 | 851 (16.1%) | 84 (14.3%) | 529 (15.7%) | 238 (18%) | 0.077 |
| ≥88 | 4421 (83.9%) | 502 (85.7%) | 2833 (84.3%) | 1086 (82%) |  |
| **Physical activity** |  |  |  |  |  |
| Low | 1180 (22.4%) | 75 (12.8%) | 620 (18.4%) | 485 (36.6%) | **<0.001** |
| Moderate | 3565 (67.6%) | 399 (68.1%) | 2393 (71.2%) | 771 (58.4%) |  |
| Good | 527 (10%) | 112 (19.1%) | 349 (10.4%) | 66 (5%) |  |
| **Smoking status** |  |  |  |  |  |
| Nonsmokers | 4995 (94.7%) | 547 (93.3%) | 3192 (94.9%) | 1256 (94.9%) | 0.072 |
| Current smokers | 107 (2%) | 9 (1.5%) | 72 (2.1%) | 26 (2%) |  |
| Past smokers | 170 (3.2%) | 30 (5.1%) | 98 (2.9%) | 42 (3.2%) |  |
| **Alcohol use** |  |  |  |  |  |
| Yes | 4 (0.1%) | 0 (0%) | 3 (0.1%) | 1 (0.1%) | 0.769 |
| No | 5268 (99.9%) | 586 (100%) | 3359 (99.9%) | 1323 (99.9%) |  |
| **Depression** | 250 (4.7%) | 33 (5.6%) | 149 (4.4%) | 68 (5.1%) | 0.333 |
| **Cardiovascular diseases** | 889 (16.9%) | 119 (20.3%) | 577 (17.2%) | 193 (14.6%) | **0.006** |
| **Hypertension** | 965 (18.3%) | 129 (22%) | 635 (18.9%) | 201 (15.2%) | **0.001** |
| **Hyperlipidemia** | 1920 (36.4%) | 209 (35.7%) | 1245 (37%) | 466 (35.2%) | 0.462 |
| **Diabetes** | 443 (8.4%) | 64 (10.9%) | 275 (8.2%) | 104 (7.9%) | 0.062 |
| Values are expressed as frequency (percent) except for age, mobile use time, and BMI, which are expressed as mean (SD)  BMI= body mass index; WC= waist circumferences | | | | | |
